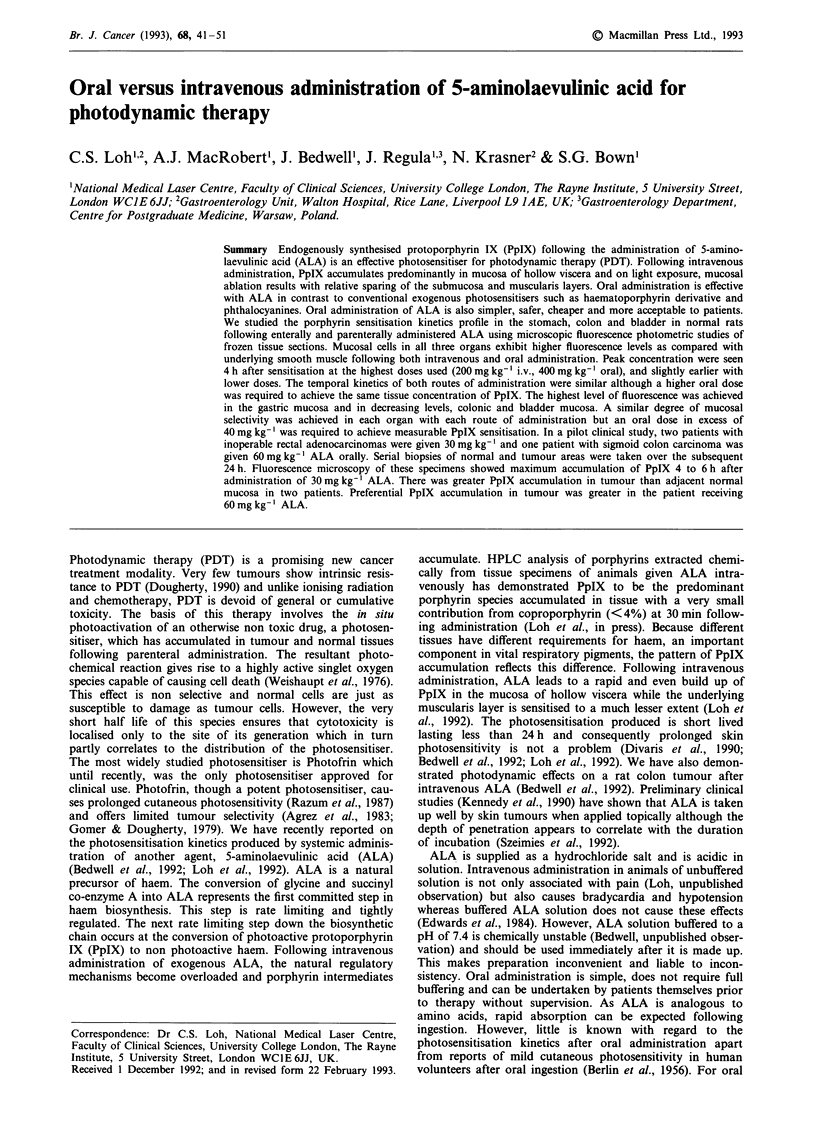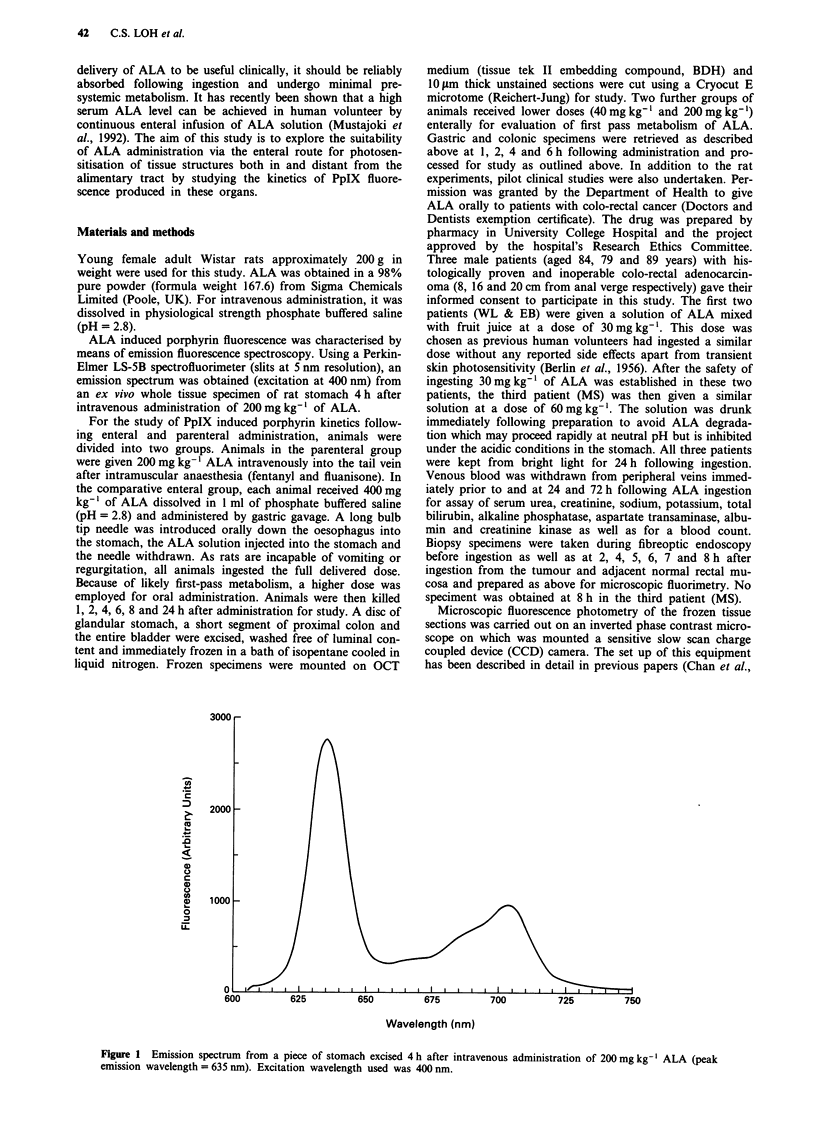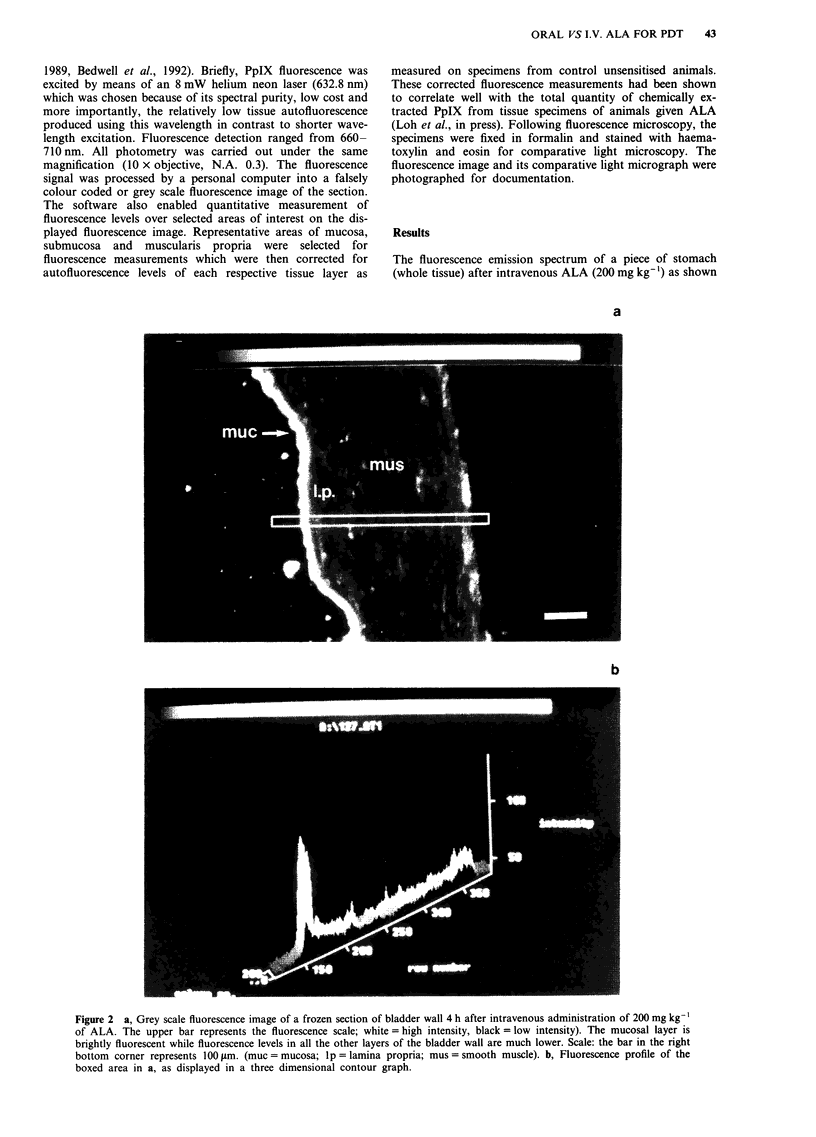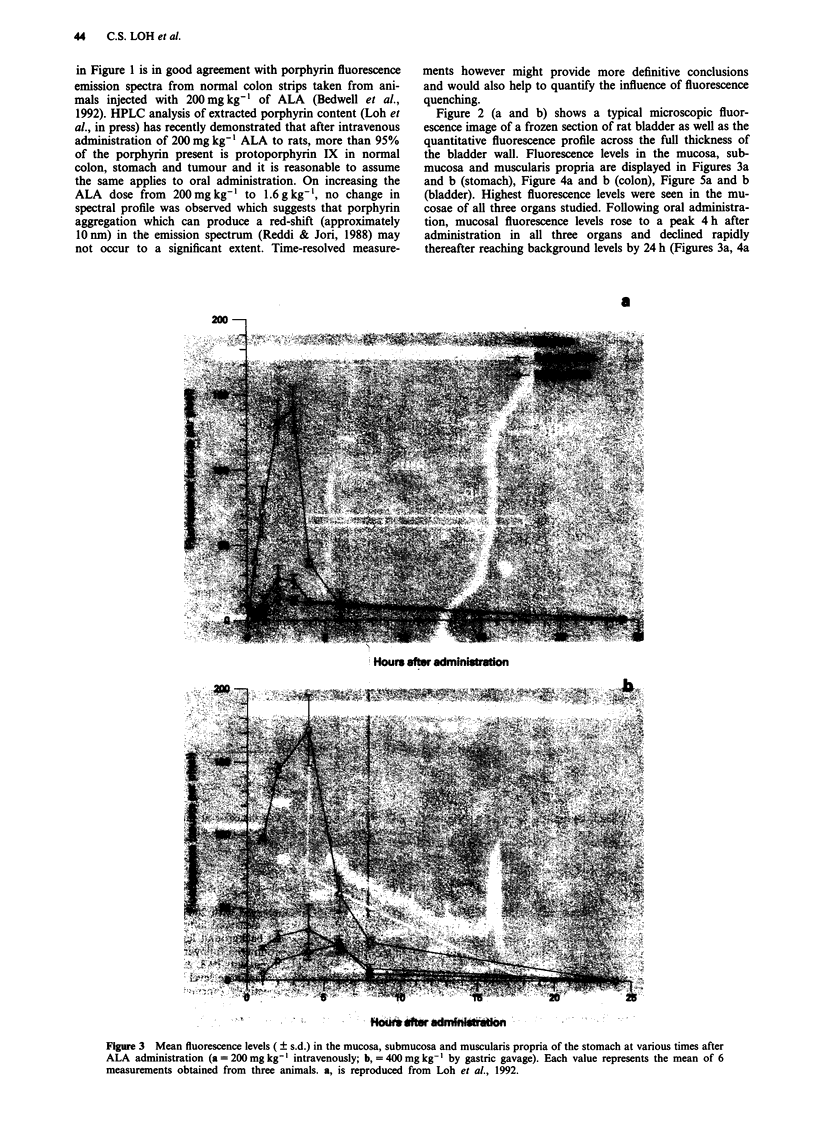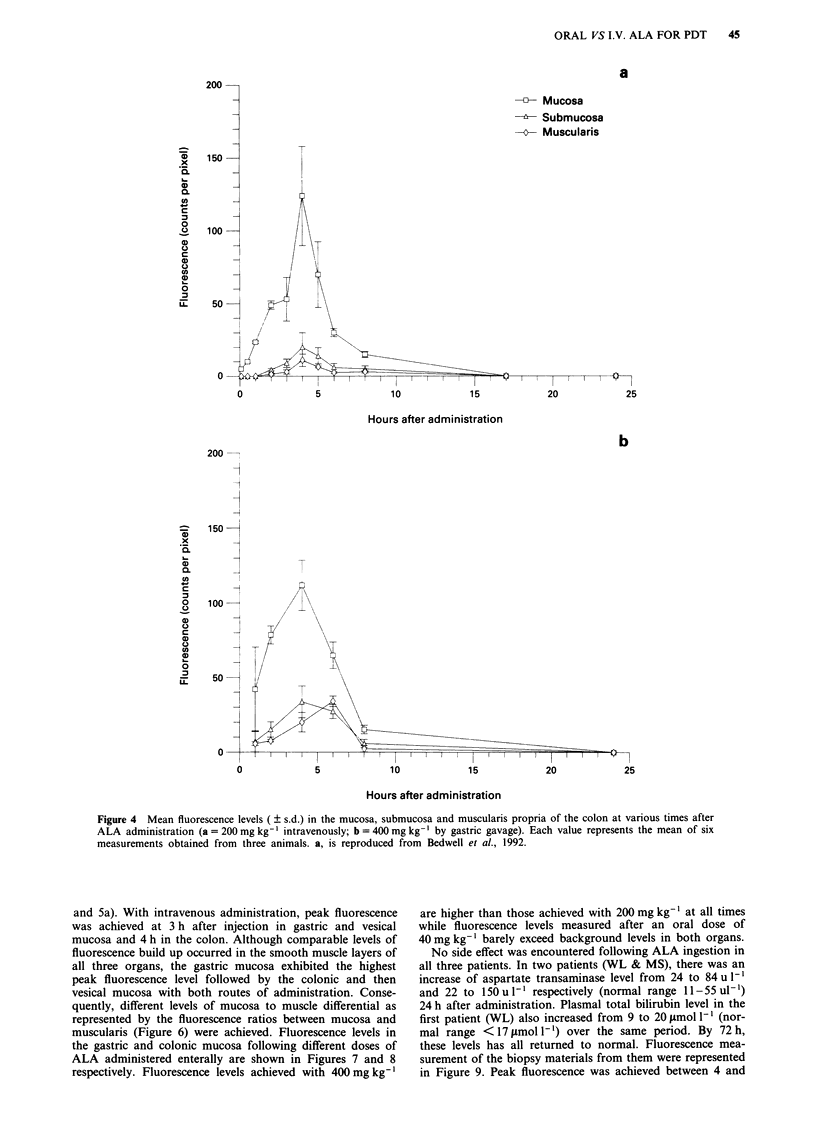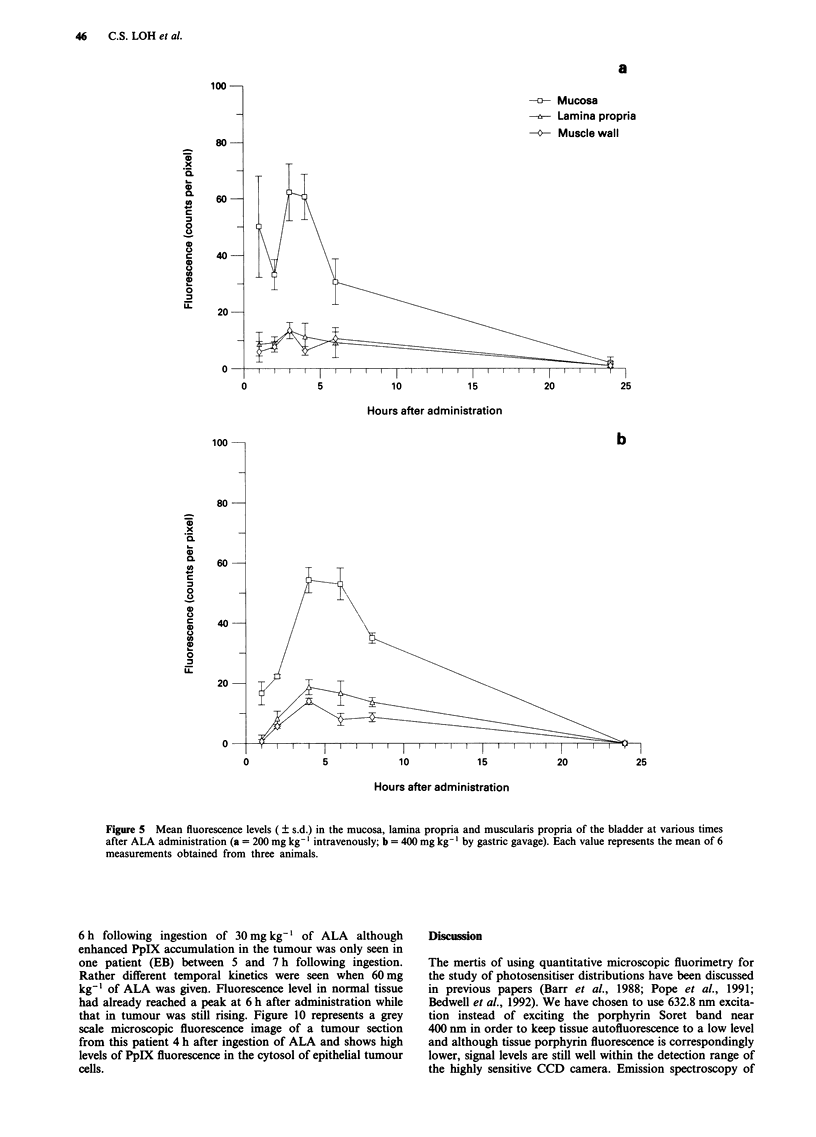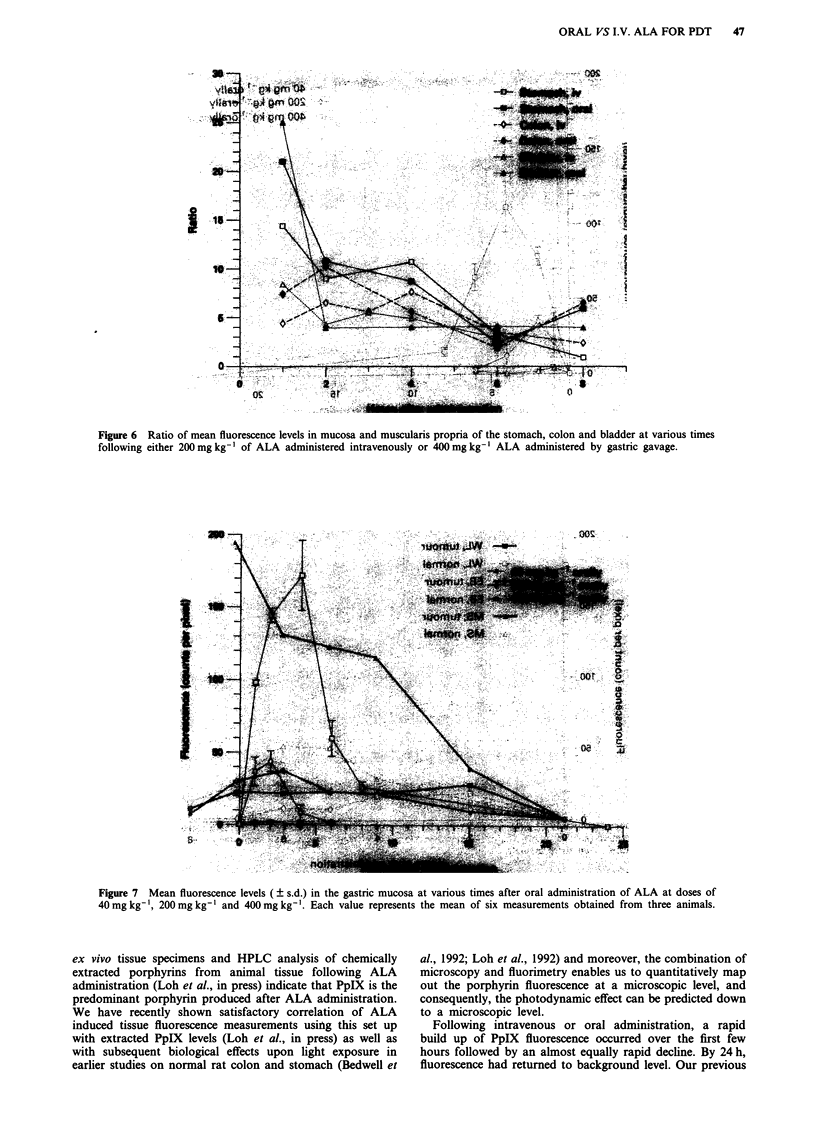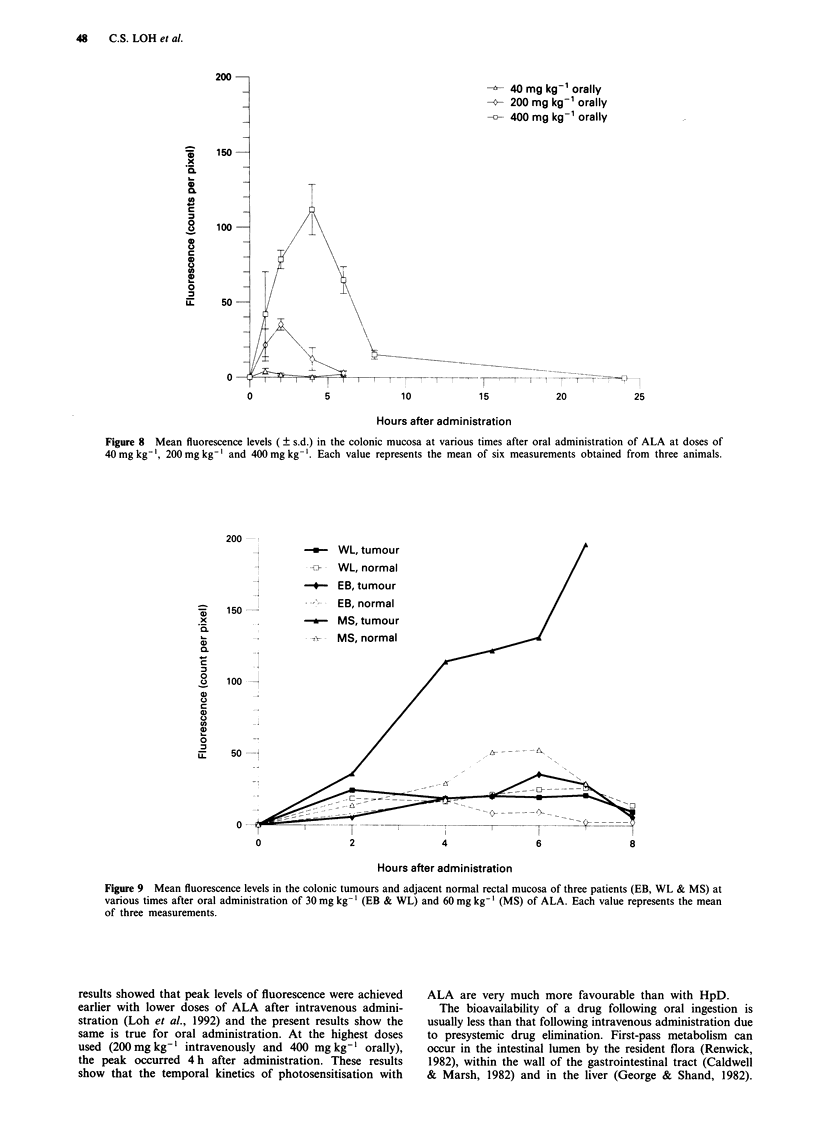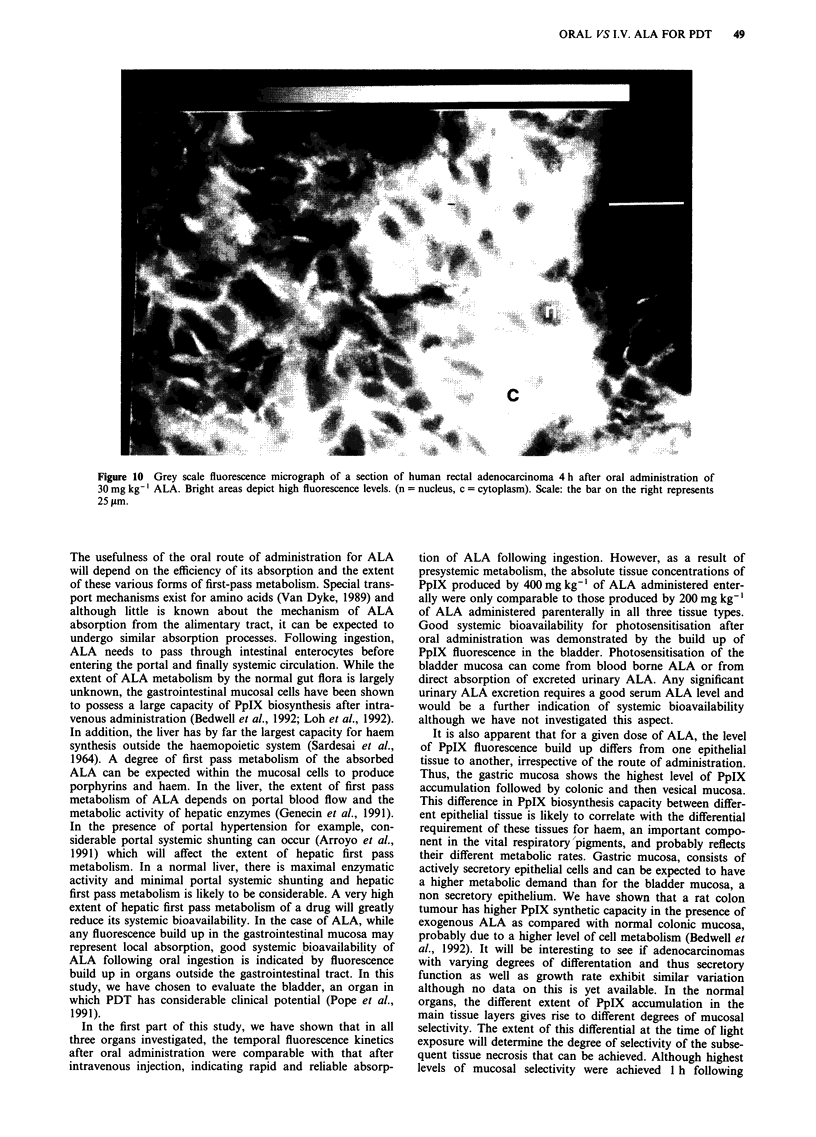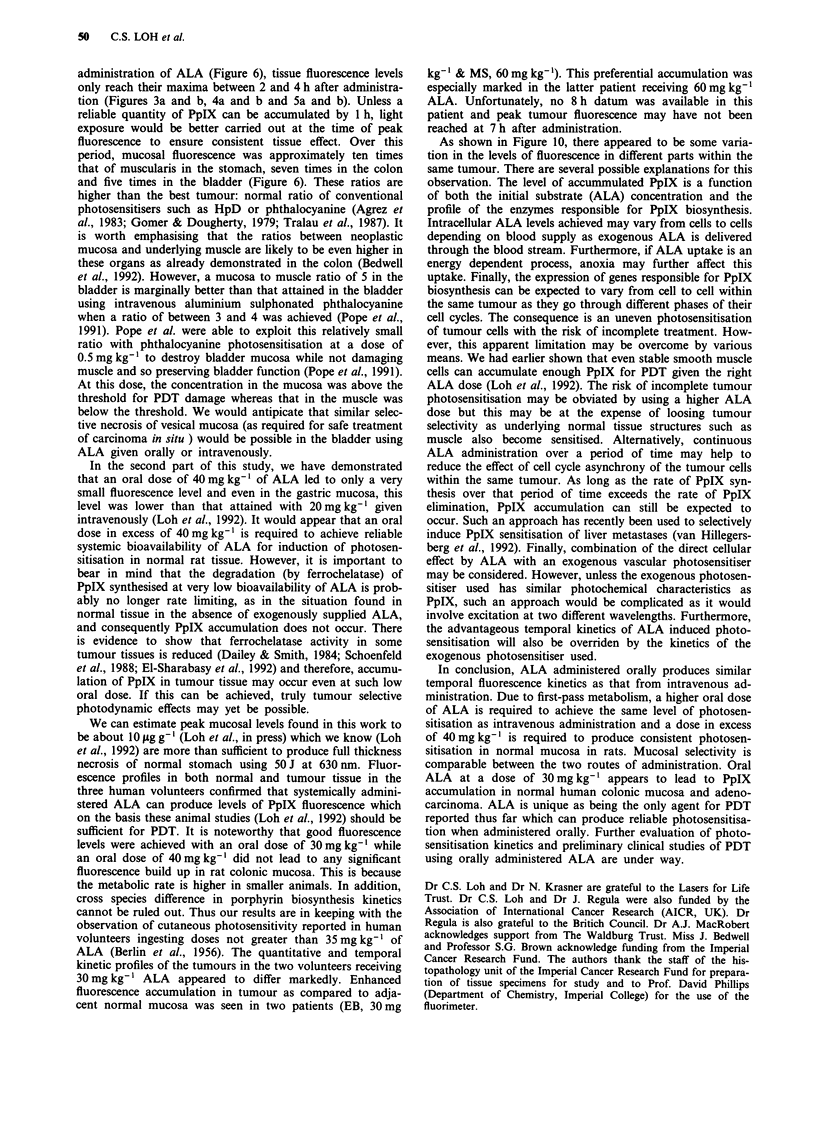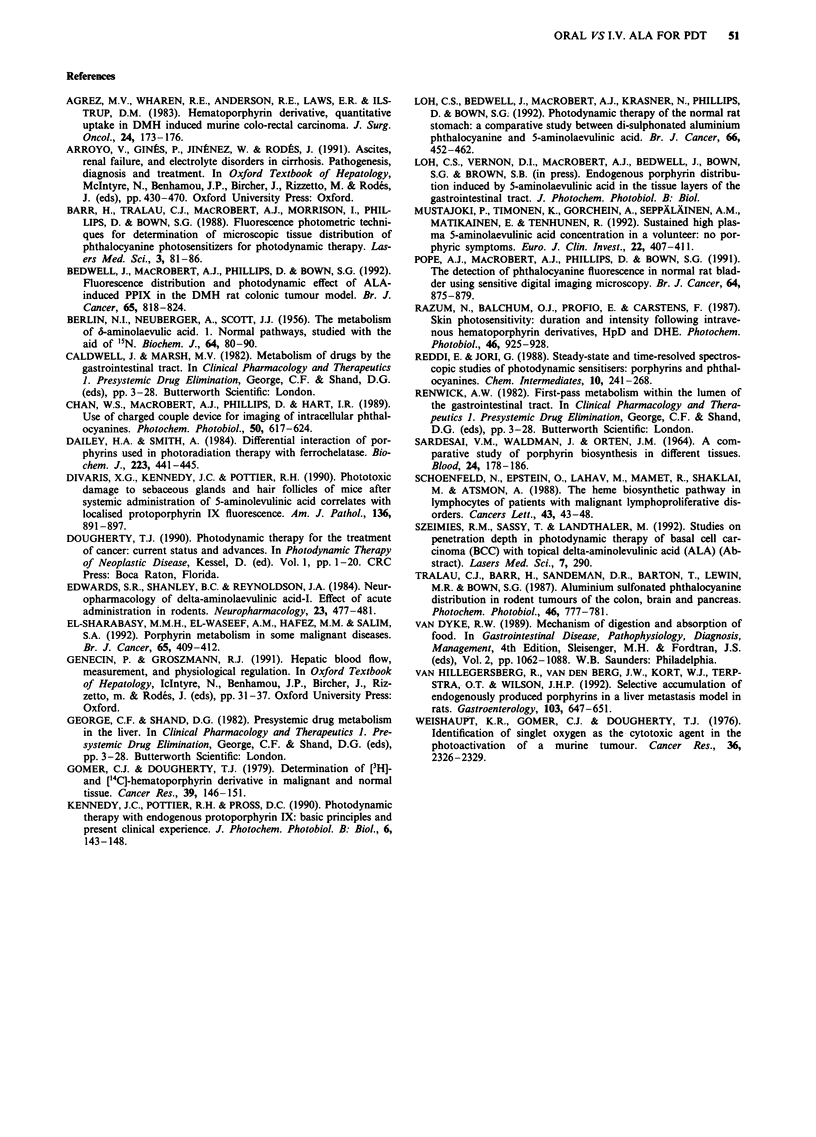# Oral versus intravenous administration of 5-aminolaevulinic acid for photodynamic therapy.

**DOI:** 10.1038/bjc.1993.284

**Published:** 1993-07

**Authors:** C. S. Loh, A. J. MacRobert, J. Bedwell, J. Regula, N. Krasner, S. G. Bown

**Affiliations:** National Medical Laser Centre, Faculty of Clinical Sciences, University College London, Rayne Institute, UK.

## Abstract

**Images:**


Br. J. Cancer (1993), 68, 41–51 © Macmillan Press Ltd., 1993

### Oral versus intravenous administration of 5-aminolaevulinic acid for photodynamic therapy

C.S. Loh[1,2], A.J. MacRobert[1], J. Bedwell[1], J. Regula[1,3], N. Krasner[2] & S.G. Bown[1]

[1]*National Medical Laser Centre, Faculty of Clinical Sciences, University College London, The Rayne Institute, 5 University Street, London WC1E 6JJ;* [2]*Gastroenterology Unit, Walton Hospital, Rice Lane, Liverpool L9 1AE, UK;* [3]*Gastroenterology Department, Centre for Postgraduate Medicine, Warsaw, Poland.*

**Summary** Endogenously synthesised protoporphyrin IX (PpIX) following the administration of 5-aminolaevulinic acid (ALA) is an effective photosensitiser for photodynamic therapy (PDT). Following intravenous administration, PpIX accumulates predominantly in mucosa of hollow viscera and on light exposure, mucosal ablation results with relative sparing of the submucosa and muscularis layers. Oral administration is effective with ALA in contrast to conventional exogenous photosensitisers such as haematoporphyrin derivative and phthalocyanines. Oral administration of ALA is also simpler, safer, cheaper and more acceptable to patients. We studied the porphyrin sensitisation kinetics profile in the stomach, colon and bladder in normal rats following enterally and parenterally administered ALA using microscopic fluorescence photometric studies of frozen tissue sections. Mucosal cells in all three organs exhibit higher fluorescence levels as compared with underlying smooth muscle following both intravenous and oral administration. Peak concentration were seen 4 h after sensitisation at the highest doses used (200 mg kg$^{-1}$ i.v., 400 mg kg$^{-1}$ oral), and slightly earlier with lower doses. The temporal kinetics of both routes of administration were similar although a higher oral dose was required to achieve the same tissue concentration of PpIX. The highest level of fluorescence was achieved in the gastric mucosa and in decreasing levels, colonic and bladder mucosa. A similar degree of mucosal selectivity was achieved in each organ with each route of administration but an oral dose in excess of 40 mg kg$^{-1}$ was required to achieve measurable PpIX sensitisation. In a pilot clinical study, two patients with inoperable rectal adenocarcinomas were given 30 mg kg$^{-1}$ and one patient with sigmoid colon carcinoma was given 60 mg kg$^{-1}$ ALA orally. Serial biopsies of normal and tumour areas were taken over the subsequent 24 h. Fluorescence microscopy of these specimens showed maximum accumulation of PpIX 4 to 6 h after administration of 30 mg kg$^{-1}$ ALA. There was greater PpIX accumulation in tumour than adjacent normal mucosa in two patients. Preferential PpIX accumulation in tumour was greater in the patient receiving 60 mg kg$^{-1}$ ALA.

Photodynamic therapy (PDT) is a promising new cancer treatment modality. Very few tumours show intrinsic resistance to PDT (Dougherty, 1990) and unlike ionising radiation and chemotherapy, PDT is devoid of general or cumulative toxicity. The basis of this therapy involves the *in situ* photoactivation of an otherwise non toxic drug, a photosensitiser, which has accumulated in tumour and normal tissues following parenteral administration. The resultant photochemical reaction gives rise to a highly active singlet oxygen species capable of causing cell death (Weishaupt *et al.*, 1976). This effect is non selective and normal cells are just as susceptible to damage as tumour cells. However, the very short half life of this species ensures that cytotoxicity is localised only to the site of its generation which in turn partly correlates to the distribution of the photosensitiser. The most widely studied photosensitiser is Photofrin which until recently, was the only photosensitiser approved for clinical use. Photofrin, though a potent photosensitiser, causes prolonged cutaneous photosensitivity (Razum *et al.*, 1987) and offers limited tumour selectivity (Agrez *et al.*, 1983; Gomer & Dougherty, 1979). We have recently reported on the photosensitisation kinetics produced by systemic administration of another agent, 5-aminolaevulinic acid (ALA) (Bedwell *et al.*, 1992; Loh *et al.*, 1992). ALA is a natural precursor of haem. The conversion of glycine and succinyl co-enzyme A into ALA represents the first committed step in haem biosynthesis. This step is rate limiting and tightly regulated. The next rate limiting step down the biosynthetic chain occurs at the conversion of photoactive protoporphyrin IX (PpIX) to non photoactive haem. Following intravenous administration of exogenous ALA, the natural regulatory mechanisms become overloaded and porphyrin intermediates accumulate. HPLC analysis of porphyrins extracted chemically from tissue specimens of animals given ALA intravenously has demonstrated PpIX to be the predominant porphyrin species accumulated in tissue with a very small contribution from coproporphyrin (<4%) at 30 min following administration (Loh *et al.*, in press). Because different tissues have different requirements for haem, an important component in vital respiratory pigments, the pattern of PpIX accumulation reflects this difference. Following intravenous administration, ALA leads to a rapid and even build up of PpIX in the mucosa of hollow viscera while the underlying muscularis layer is sensitised to a much lesser extent (Loh *et al.*, 1992). The photosensitisation produced is short lived lasting less than 24 h and consequently prolonged skin photosensitivity is not a problem (Divaris *et al.*, 1990; Bedwell *et al.*, 1992; Loh *et al.*, 1992). We have also demonstrated photodynamic effects on a rat colon tumour after intravenous ALA (Bedwell *et al.*, 1992). Preliminary clinical studies (Kennedy *et al.*, 1990) have shown that ALA is taken up well by skin tumours when applied topically although the depth of penetration appears to correlate with the duration of incubation (Szeimies *et al.*, 1992).

ALA is supplied as a hydrochloride salt and is acidic in solution. Intravenous administration in animals of unbuffered solution is not only associated with pain (Loh, unpublished observation) but also causes bradycardia and hypotension whereas buffered ALA solution does not cause these effects (Edwards *et al.*, 1984). However, ALA solution buffered to a pH of 7.4 is chemically unstable (Bedwell, unpublished observation) and should be used immediately after it is made up. This makes preparation inconvenient and liable to inconsistency. Oral administration is simple, does not require full buffering and can be undertaken by patients themselves prior to therapy without supervision. As ALA is analogous to amino acids, rapid absorption can be expected following ingestion. However, little is known with regard to the photosensitisation kinetics after oral administration apart from reports of mild cutaneous photosensitivity in human volunteers after oral ingestion (Berlin *et al.*, 1956). For oral

Correspondence: Dr C.S. Loh, National Medical Laser Centre, Faculty of Clinical Sciences, University College London, The Rayne Institute, 5 University Street, London WC1E 6JJ, UK.
Received 1 December 1992; and in revised form 22 February 1993.

delivery of ALA to be useful clinically, it should be reliably absorbed following ingestion and undergo minimal pre-systemic metabolism. It has recently been shown that a high serum ALA level can be achieved in human volunteer by continuous enteral infusion of ALA solution (Mustajoki *et al.*, 1992). The aim of this study is to explore the suitability of ALA administration via the enteral route for photosensitisation of tissue structures both in and distant from the alimentary tract by studying the kinetics of PpIX fluorescence produced in these organs.

### Materials and methods

Young female adult Wistar rats approximately 200 g in weight were used for this study. ALA was obtained in a 98% pure powder (formula weight 167.6) from Sigma Chemicals Limited (Poole, UK). For intravenous administration, it was dissolved in physiological strength phosphate buffered saline (pH = 2.8).

ALA induced porphyrin fluorescence was characterised by means of emission fluorescence spectroscopy. Using a Perkin-Elmer LS-5B spectrofluorimeter (slits at 5 nm resolution), an emission spectrum was obtained (excitation at 400 nm) from an *ex vivo* whole tissue specimen of rat stomach 4 h after intravenous administration of 200 mg $kg^{-1}$ of ALA.

For the study of PpIX induced porphyrin kinetics following enteral and parenteral administration, animals were divided into two groups. Animals in the parenteral group were given 200 mg $kg^{-1}$ ALA intravenously into the tail vein after intramuscular anaesthesia (fentanyl and fluanisone). In the comparative enteral group, each animal received 400 mg $kg^{-1}$ of ALA dissolved in 1 ml of phosphate buffered saline (pH = 2.8) and administered by gastric gavage. A long bulb tip needle was introduced orally down the oesophagus into the stomach, the ALA solution injected into the stomach and the needle withdrawn. As rats are incapable of vomiting or regurgitation, all animals ingested the full delivered dose. Because of likely first-pass metabolism, a higher dose was employed for oral administration. Animals were then killed 1, 2, 4, 6, 8 and 24 h after administration for study. A disc of glandular stomach, a short segment of proximal colon and the entire bladder were excised, washed free of luminal content and immediately frozen in a bath of isopentane cooled in liquid nitrogen. Frozen specimens were mounted on OCT medium (tissue tek II embedding compound, BDH) and 10 $\mu m$ thick unstained sections were cut using a Cryocut E microtome (Reichert-Jung) for study. Two further groups of animals received lower doses (40 mg $kg^{-1}$ and 200 mg $kg^{-1}$) enterally for evaluation of first pass metabolism of ALA. Gastric and colonic specimens were retrieved as described above at 1, 2, 4 and 6 h following administration and processed for study as outlined above. In addition to the rat experiments, pilot clinical studies were also undertaken. Permission was granted by the Department of Health to give ALA orally to patients with colo-rectal cancer (Doctors and Dentists exemption certificate). The drug was prepared by pharmacy in University College Hospital and the project approved by the hospital's Research Ethics Committee. Three male patients (aged 84, 79 and 89 years) with histologically proven and inoperable colo-rectal adenocarcinoma (8, 16 and 20 cm from anal verge respectively) gave their informed consent to participate in this study. The first two patients (WL & EB) were given a solution of ALA mixed with fruit juice at a dose of 30 mg $kg^{-1}$. This dose was chosen as previous human volunteers had ingested a similar dose without any reported side effects apart from transient skin photosensitivity (Berlin *et al.*, 1956). After the safety of ingesting 30 mg $kg^{-1}$ of ALA was established in these two patients, the third patient (MS) was then given a similar solution at a dose of 60 mg $kg^{-1}$. The solution was drunk immediately following preparation to avoid ALA degradation which may proceed rapidly at neutral pH but is inhibited under the acidic conditions in the stomach. All three patients were kept from bright light for 24 h following ingestion. Venous blood was withdrawn from peripheral veins immediately prior to and at 24 and 72 h following ALA ingestion for assay of serum urea, creatinine, sodium, potassium, total bilirubin, alkaline phosphatase, aspartate transaminase, albumin and creatinine kinase as well as for a blood count. Biopsy specimens were taken during fibreoptic endoscopy before ingestion as well as at 2, 4, 5, 6, 7 and 8 h after ingestion from the tumour and adjacent normal rectal mucosa and prepared as above for microscopic fluorimetry. No speciment was obtained at 8 h in the third patient (MS).

Microscopic fluorescence photometry of the frozen tissue sections was carried out on an inverted phase contrast microscope on which was mounted a sensitive slow scan charge coupled device (CCD) camera. The set up of this equipment has been described in detail in previous papers (Chan *et al.*,

**Figure 1** Emission spectrum from a piece of stomach excised 4 h after intravenous administration of 200 mg $kg^{-1}$ ALA (peak emission wavelength = 635 nm). Excitation wavelength used was 400 nm.

1989, Bedwell *et al.*, 1992). Briefly, PpIX fluorescence was excited by means of an 8 mW helium neon laser (632.8 nm) which was chosen because of its spectral purity, low cost and more importantly, the relatively low tissue autofluorescence produced using this wavelength in contrast to shorter wavelength excitation. Fluorescence detection ranged from 660–710 nm. All photometry was carried out under the same magnification (10 × objective, N.A. 0.3). The fluorescence signal was processed by a personal computer into a falsely colour coded or grey scale fluorescence image of the section. The software also enabled quantitative measurement of fluorescence levels over selected areas of interest on the displayed fluorescence image. Representative areas of mucosa, submucosa and muscularis propria were selected for fluorescence measurements which were then corrected for autofluorescence levels of each respective tissue layer as measured on specimens from control unsensitised animals. These corrected fluorescence measurements had been shown to correlate well with the total quantity of chemically extracted PpIX from tissue specimens of animals given ALA (Loh *et al.*, in press). Following fluorescence microscopy, the specimens were fixed in formalin and stained with haematoxylin and eosin for comparative light microscopy. The fluorescence image and its comparative light micrograph were photographed for documentation.

### Results

The fluorescence emission spectrum of a piece of stomach (whole tissue) after intravenous ALA (200 mg $kg^{-1}$) as shown

**Figure 2** **a**, Grey scale fluorescence image of a frozen section of bladder wall 4 h after intravenous administration of 200 mg $kg^{-1}$ of ALA. The upper bar represents the fluorescence scale; white = high intensity, black = low intensity). The mucosal layer is brightly fluorescent while fluorescence levels in all the other layers of the bladder wall are much lower. Scale: the bar in the right bottom corner represents 100 µm. (muc = mucosa; 1p = lamina propria; mus = smooth muscle). **b**, Fluorescence profile of the boxed area in **a**, as displayed in a three dimensional contour graph.

in Figure 1 is in good agreement with porphyrin fluorescence emission spectra from normal colon strips taken from animals injected with 200 mg kg$^{-1}$ of ALA (Bedwell *et al.*, 1992). HPLC analysis of extracted porphyrin content (Loh *et al.*, in press) has recently demonstrated that after intravenous administration of 200 mg kg$^{-1}$ ALA to rats, more than 95% of the porphyrin present is protoporphyrin IX in normal colon, stomach and tumour and it is reasonable to assume the same applies to oral administration. On increasing the ALA dose from 200 mg kg$^{-1}$ to 1.6 g kg$^{-1}$, no change in spectral profile was observed which suggests that porphyrin aggregation which can produce a red-shift (approximately 10 nm) in the emission spectrum (Reddi & Jori, 1988) may not occur to a significant extent. Time-resolved measurements however might provide more definitive conclusions and would also help to quantify the influence of fluorescence quenching.

Figure 2 (a and b) shows a typical microscopic fluorescence image of a frozen section of rat bladder as well as the quantitative fluorescence profile across the full thickness of the bladder wall. Fluorescence levels in the mucosa, submucosa and muscularis propria are displayed in Figures 3a and b (stomach), Figure 4a and b (colon), Figure 5a and b (bladder). Highest fluorescence levels were seen in the mucosae of all three organs studied. Following oral administration, mucosal fluorescence levels rose to a peak 4 h after administration in all three organs and declined rapidly thereafter reaching background levels by 24 h (Figures 3a, 4a

**Figure 3** Mean fluorescence levels (± s.d.) in the mucosa, submucosa and muscularis propria of the stomach at various times after ALA administration (**a** = 200 mg kg$^{-1}$ intravenously; **b**, = 400 mg kg$^{-1}$ by gastric gavage). Each value represents the mean of 6 measurements obtained from three animals. **a**, is reproduced from Loh *et al.*, 1992.

**Figure 4** Mean fluorescence levels ( ± s.d.) in the mucosa, submucosa and muscularis propria of the colon at various times after ALA administration (**a** = 200 mg kg$^{-1}$ intravenously; **b** = 400 mg kg$^{-1}$ by gastric gavage). Each value represents the mean of six measurements obtained from three animals. **a**, is reproduced from Bedwell *et al.*, 1992.

and 5a). With intravenous administration, peak fluorescence was achieved at 3 h after injection in gastric and vesical mucosa and 4 h in the colon. Although comparable levels of fluorescence build up occurred in the smooth muscle layers of all three organs, the gastric mucosa exhibited the highest peak fluorescence level followed by the colonic and then vesical mucosa with both routes of administration. Consequently, different levels of mucosa to muscle differential as represented by the fluorescence ratios between mucosa and muscularis (Figure 6) were achieved. Fluorescence levels in the gastric and colonic mucosa following different doses of ALA administered enterally are shown in Figures 7 and 8 respectively. Fluorescence levels achieved with 400 mg kg$^{-1}$ are higher than those achieved with 200 mg kg$^{-1}$ at all times while fluorescence levels measured after an oral dose of 40 mg kg$^{-1}$ barely exceed background levels in both organs.

No side effect was encountered following ALA ingestion in all three patients. In two patients (WL & MS), there was an increase of aspartate transaminase level from 24 to 84 u l$^{-1}$ and 22 to 150 u l$^{-1}$ respectively (normal range 11–55 ul$^{-1}$) 24 h after administration. Plasmal total bilirubin level in the first patient (WL) also increased from 9 to 20 μmol l$^{-1}$ (normal range < 17 μmol l$^{-1}$) over the same period. By 72 h, these levels has all returned to normal. Fluorescence measurement of the biopsy materials from them were represented in Figure 9. Peak fluorescence was achieved between 4 and

**Figure 5** Mean fluorescence levels ( ± s.d.) in the mucosa, lamina propria and muscularis propria of the bladder at various times after ALA administration (**a** = 200 mg kg$^{-1}$ intravenously; **b** = 400 mg kg$^{-1}$ by gastric gavage). Each value represents the mean of 6 measurements obtained from three animals.

6 h following ingestion of 30 mg kg$^{-1}$ of ALA although enhanced PpIX accumulation in the tumour was only seen in one patient (EB) between 5 and 7 h following ingestion. Rather different temporal kinetics were seen when 60 mg kg$^{-1}$ of ALA was given. Fluorescence level in normal tissue had already reached a peak at 6 h after administration while that in tumour was still rising. Figure 10 represents a grey scale microscopic fluorescence image of a tumour section from this patient 4 h after ingestion of ALA and shows high levels of PpIX fluorescence in the cytosol of epithelial tumour cells.

### Discussion

The mertis of using quantitative microscopic fluorimetry for the study of photosensitiser distributions have been discussed in previous papers (Barr *et al.*, 1988; Pope *et al.*, 1991; Bedwell *et al.*, 1992). We have chosen to use 632.8 nm excitation instead of exciting the porphyrin Soret band near 400 nm in order to keep tissue autofluorescence to a low level and although tissue porphyrin fluorescence is correspondingly lower, signal levels are still well within the detection range of the highly sensitive CCD camera. Emission spectroscopy of

**Figure 6** Ratio of mean fluorescence levels in mucosa and muscularis propria of the stomach, colon and bladder at various times following either 200 mg $kg^{-1}$ of ALA administered intravenously or 400 mg $kg^{-1}$ ALA administered by gastric gavage.

**Figure 7** Mean fluorescence levels ( ± s.d.) in the gastric mucosa at various times after oral administration of ALA at doses of 40 mg $kg^{-1}$, 200 mg $kg^{-1}$ and 400 mg $kg^{-1}$. Each value represents the mean of six measurements obtained from three animals.

*ex vivo* tissue specimens and HPLC analysis of chemically extracted porphyrins from animal tissue following ALA administration (Loh *et al.*, in press) indicate that PpIX is the predominant porphyrin produced after ALA administration. We have recently shown satisfactory correlation of ALA induced tissue fluorescence measurements using this set up with extracted PpIX levels (Loh *et al.*, in press) as well as with subsequent biological effects upon light exposure in earlier studies on normal rat colon and stomach (Bedwell *et al.*, 1992; Loh *et al.*, 1992) and moreover, the combination of microscopy and fluorimetry enables us to quantitatively map out the porphyrin fluorescence at a microscopic level, and consequently, the photodynamic effect can be predicted down to a microscopic level.

Following intravenous or oral administration, a rapid build up of PpIX fluorescence occurred over the first few hours followed by an almost equally rapid decline. By 24 h, fluorescence had returned to background level. Our previous

**Figure 8** Mean fluorescence levels ( $\pm$ s.d.) in the colonic mucosa at various times after oral administration of ALA at doses of 40 mg $kg^{-1}$, 200 mg $kg^{-1}$ and 400 mg $kg^{-1}$. Each value represents the mean of six measurements obtained from three animals.

**Figure 9** Mean fluorescence levels in the colonic tumours and adjacent normal rectal mucosa of three patients (EB, WL & MS) at various times after oral administration of 30 mg $kg^{-1}$ (EB & WL) and 60 mg $kg^{-1}$ (MS) of ALA. Each value represents the mean of three measurements.

results showed that peak levels of fluorescence were achieved earlier with lower doses of ALA after intravenous administration (Loh *et al.*, 1992) and the present results show the same is true for oral administration. At the highest doses used (200 mg $kg^{-1}$ intravenously and 400 mg $kg^{-1}$ orally), the peak occurred 4 h after administration. These results show that the temporal kinetics of photosensitisation with ALA are very much more favourable than with HpD.

The bioavailability of a drug following oral ingestion is usually less than that following intravenous administration due to presystemic drug elimination. First-pass metabolism can occur in the intestinal lumen by the resident flora (Renwick, 1982), within the wall of the gastrointestinal tract (Caldwell & Marsh, 1982) and in the liver (George & Shand, 1982).

**Figure 10** Grey scale fluorescence micrograph of a section of human rectal adenocarcinoma 4 h after oral administration of 30 mg $kg^{-1}$ ALA. Bright areas depict high fluorescence levels. (n = nucleus, c = cytoplasm). Scale: the bar on the right represents 25 $\mu$m.

The usefulness of the oral route of administration for ALA will depend on the efficiency of its absorption and the extent of these various forms of first-pass metabolism. Special transport mechanisms exist for amino acids (Van Dyke, 1989) and although little is known about the mechanism of ALA absorption from the alimentary tract, it can be expected to undergo similar absorption processes. Following ingestion, ALA needs to pass through intestinal enterocytes before entering the portal and finally systemic circulation. While the extent of ALA metabolism by the normal gut flora is largely unknown, the gastrointestinal mucosal cells have been shown to possess a large capacity of PpIX biosynthesis after intravenous administration (Bedwell *et al.*, 1992; Loh *et al.*, 1992). In addition, the liver has by far the largest capacity for haem synthesis outside the haemopoietic system (Sardesai *et al.*, 1964). A degree of first pass metabolism of the absorbed ALA can be expected within the mucosal cells to produce porphyrins and haem. In the liver, the extent of first pass metabolism of ALA depends on portal blood flow and the metabolic activity of hepatic enzymes (Genecin *et al.*, 1991). In the presence of portal hypertension for example, considerable portal systemic shunting can occur (Arroyo *et al.*, 1991) which will affect the extent of hepatic first pass metabolism. In a normal liver, there is maximal enzymatic activity and minimal portal systemic shunting and hepatic first pass metabolism is likely to be considerable. A very high extent of hepatic first pass metabolism of a drug will greatly reduce its systemic bioavailability. In the case of ALA, while any fluorescence build up in the gastrointestinal mucosa may represent local absorption, good systemic bioavailability of ALA following oral ingestion is indicated by fluorescence build up in organs outside the gastrointestinal tract. In this study, we have chosen to evaluate the bladder, an organ in which PDT has considerable clinical potential (Pope *et al.*, 1991).

In the first part of this study, we have shown that in all three organs investigated, the temporal fluorescence kinetics after oral administration were comparable with that after intravenous injection, indicating rapid and reliable absorption of ALA following ingestion. However, as a result of presystemic metabolism, the absolute tissue concentrations of PpIX produced by 400 mg $kg^{-1}$ of ALA administered enterally were only comparable to those produced by 200 mg $kg^{-1}$ of ALA administered parenterally in all three tissue types. Good systemic bioavailability for photosensitisation after oral administration was demonstrated by the build up of PpIX fluorescence in the bladder. Photosensitisation of the bladder mucosa can come from blood borne ALA or from direct absorption of excreted urinary ALA. Any significant urinary ALA excretion requires a good serum ALA level and would be a further indication of systemic bioavailability although we have not investigated this aspect.

It is also apparent that for a given dose of ALA, the level of PpIX fluorescence build up differs from one epithelial tissue to another, irrespective of the route of administration. Thus, the gastric mucosa shows the highest level of PpIX accumulation followed by colonic and then vesical mucosa. This difference in PpIX biosynthesis capacity between different epithelial tissue is likely to correlate with the differential requirement of these tissues for haem, an important component in the vital respiratory pigments, and probably reflects their different metabolic rates. Gastric mucosa, consists of actively secretory epithelial cells and can be expected to have a higher metabolic demand than for the bladder mucosa, a non secretory epithelium. We have shown that a rat colon tumour has higher PpIX synthetic capacity in the presence of exogenous ALA as compared with normal colonic mucosa, probably due to a higher level of cell metabolism (Bedwell *et al.*, 1992). It will be interesting to see if adenocarcinomas with varying degrees of differentation and thus secretory function as well as growth rate exhibit similar variation although no data on this is yet available. In the normal organs, the different extent of PpIX accumulation in the main tissue layers gives rise to different degrees of mucosal selectivity. The extent of this differential at the time of light exposure will determine the degree of selectivity of the subsequent tissue necrosis that can be achieved. Although highest levels of mucosal selectivity were achieved 1 h following administration of ALA (Figure 6), tissue fluorescence levels only reach their maxima between 2 and 4 h after administration (Figures 3a and b, 4a and b and 5a and b). Unless a reliable quantity of PpIX can be accumulated by 1 h, light exposure would be better carried out at the time of peak fluorescence to ensure consistent tissue effect. Over this period, mucosal fluorescence was approximately ten times that of muscularis in the stomach, seven times in the colon and five times in the bladder (Figure 6). These ratios are higher than the best tumour: normal ratio of conventional photosensitisers such as HpD or phthalocyanine (Agrez et al., 1983; Gomer & Dougherty, 1979; Tralau et al., 1987). It is worth emphasising that the ratios between neoplastic mucosa and underlying muscle are likely to be even higher in these organs as already demonstrated in the colon (Bedwell et al., 1992). However, a mucosa to muscle ratio of 5 in the bladder is marginally better than that attained in the bladder using intravenous aluminium sulphonated phthalocyanine when a ratio of between 3 and 4 was achieved (Pope et al., 1991). Pope et al. were able to exploit this relatively small ratio with phthalocyanine photosensitisation at a dose of 0.5 mg $kg^{-1}$ to destroy bladder mucosa while not damaging muscle and so preserving bladder function (Pope et al., 1991). At this dose, the concentration in the mucosa was above the threshold for PDT damage whereas that in the muscle was below the threshold. We would antipicate that similar selective necrosis of vesical mucosa (as required for safe treatment of carcinoma *in situ* ) would be possible in the bladder using ALA given orally or intravenously.

In the second part of this study, we have demonstrated that an oral dose of 40 mg $kg^{-1}$ of ALA led to only a very small fluorescence level and even in the gastric mucosa, this level was lower than that attained with 20 mg $kg^{-1}$ given intravenously (Loh et al., 1992). It would appear that an oral dose in excess of 40 mg $kg^{-1}$ is required to achieve reliable systemic bioavailability of ALA for induction of photosensitisation in normal rat tissue. However, it is important to bear in mind that the degradation (by ferrochelatase) of PpIX synthesised at very low bioavailability of ALA is probably no longer rate limiting, as in the situation found in normal tissue in the absence of exogenously supplied ALA, and consequently PpIX accumulation does not occur. There is evidence to show that ferrochelatase activity in some tumour tissues is reduced (Dailey & Smith, 1984; Schoenfeld et al., 1988; El-Sharabasy et al., 1992) and therefore, accumulation of PpIX in tumour tissue may occur even at such low oral dose. If this can be achieved, truly tumour selective photodynamic effects may yet be possible.

We can estimate peak mucosal levels found in this work to be about 10 μg $g^{-1}$ (Loh et al., in press) which we know (Loh et al., 1992) are more than sufficient to produce full thickness necrosis of normal stomach using 50 J at 630 nm. Fluorescence profiles in both normal and tumour tissue in the three human volunteers confirmed that systemically administered ALA can produce levels of PpIX fluorescence which on the basis these animal studies (Loh et al., 1992) should be sufficient for PDT. It is noteworthy that good fluorescence levels were achieved with an oral dose of 30 mg $kg^{-1}$ while an oral dose of 40 mg $kg^{-1}$ did not lead to any significant fluorescence build up in rat colonic mucosa. This is because the metabolic rate is higher in smaller animals. In addition, cross species difference in porphyrin biosynthesis kinetics cannot be ruled out. Thus our results are in keeping with the observation of cutaneous photosensitivity reported in human volunteers ingesting doses not greater than 35 mg $kg^{-1}$ of ALA (Berlin et al., 1956). The quantitative and temporal kinetic profiles of the tumours in the two volunteers receiving 30 mg $kg^{-1}$ ALA appeared to differ markedly. Enhanced fluorescence accumulation in tumour as compared to adjacent normal mucosa was seen in two patients (EB, 30 mg $kg^{-1}$ & MS, 60 mg $kg^{-1}$). This preferential accumulation was especially marked in the latter patient receiving 60 mg $kg^{-1}$ ALA. Unfortunately, no 8 h datum was available in this patient and peak tumour fluorescence may have not been reached at 7 h after administration.

As shown in Figure 10, there appeared to be some variation in the levels of fluorescence in different parts within the same tumour. There are several possible explanations for this observation. The level of accummulated PpIX is a function of both the initial substrate (ALA) concentration and the profile of the enzymes responsible for PpIX biosynthesis. Intracellular ALA levels achieved may vary from cells to cells depending on blood supply as exogenous ALA is delivered through the blood stream. Furthermore, if ALA uptake is an energy dependent process, anoxia may further affect this uptake. Finally, the expression of genes responsible for PpIX biosynthesis can be expected to vary from cell to cell within the same tumour as they go through different phases of their cell cycles. The consequence is an uneven photosensitisation of tumour cells with the risk of incomplete treatment. However, this apparent limitation may be overcome by various means. We had earlier shown that even stable smooth muscle cells can accumulate enough PpIX for PDT given the right ALA dose (Loh et al., 1992). The risk of incomplete tumour photosensitisation may be obviated by using a higher ALA dose but this may be at the expense of loosing tumour selectivity as underlying normal tissue structures such as muscle also become sensitised. Alternatively, continuous ALA administration over a period of time may help to reduce the effect of cell cycle asynchrony of the tumour cells within the same tumour. As long as the rate of PpIX synthesis over that period of time exceeds the rate of PpIX elimination, PpIX accumulation can still be expected to occur. Such an approach has recently been used to selectively induce PpIX sensitisation of liver metastases (van Hillegersberg et al., 1992). Finally, combination of the direct cellular effect by ALA with an exogenous vascular photosensitiser may be considered. However, unless the exogenous photosensitiser used has similar photochemical characteristics as PpIX, such an approach would be complicated as it would involve excitation at two different wavelengths. Furthermore, the advantageous temporal kinetics of ALA induced photosensitisation will also be overriden by the kinetics of the exogenous photosensitiser used.

In conclusion, ALA administered orally produces similar temporal fluorescence kinetics as that from intravenous administration. Due to first-pass metabolism, a higher oral dose of ALA is required to achieve the same level of photosensitisation as intravenous administration and a dose in excess of 40 mg $kg^{-1}$ is required to produce consistent photosensitisation in normal mucosa in rats. Mucosal selectivity is comparable between the two routes of administration. Oral ALA at a dose of 30 mg $kg^{-1}$ appears to lead to PpIX accumulation in normal human colonic mucosa and adenocarcinoma. ALA is unique as being the only agent for PDT reported thus far which can produce reliable photosensitisation when administered orally. Further evaluation of photosensitisation kinetics and preliminary clinical studies of PDT using orally administered ALA are under way.

Dr C.S. Loh and Dr N. Krasner are grateful to the Lasers for Life Trust. Dr C.S. Loh and Dr J. Regula were also funded by the Association of International Cancer Research (AICR, UK). Dr Regula is also grateful to the Britich Council. Dr A.J. MacRobert acknowledges support from The Waldburg Trust. Miss J. Bedwell and Professor S.G. Brown acknowledge funding from the Imperial Cancer Research Fund. The authors thank the staff of the histopathology unit of the Imperial Cancer Research Fund for preparation of tissue specimens for study and to Prof. David Phillips (Department of Chemistry, Imperial College) for the use of the fluorimeter.

### References

AGREZ, M.V., WHAREN, R.E., ANDERSON, R.E., LAWS, E.R. & ILSTRUP, D.M. (1983). Hematoporphyrin derivative, quantitative uptake in DMH induced murine colo-rectal carcinoma. *J. Surg. Oncol.*, **24**, 173–176.

ARROYO, V., GINÉS, P., JINÉNEZ, W. & RODÉS, J. (1991). Ascites, renal failure, and electrolyte disorders in cirrhosis. Pathogenesis, diagnosis and treatment. In *Oxford Textbook of Hepatology*, McIntyre, N., Benhamou, J.P., Bircher, J., Rizzetto, M. & Rodés, J. (eds), pp. 430–470. Oxford University Press: Oxford.

BARR, H., TRALAU, C.J., MACROBERT, A.J., MORRISON, I., PHILLIPS, D. & BOWN, S.G. (1988). Fluorescence photometric techniques for determination of microscopic tissue distribution of phthalocyanine photosensitizers for photodynamic therapy. *Lasers Med. Sci.*, **3**, 81–86.

BEDWELL, J., MACROBERT, A.J., PHILLIPS, D. & BOWN, S.G. (1992). Fluorescence distribution and photodynamic effect of ALA-induced PPIX in the DMH rat colonic tumour model. *Br. J. Cancer*, **65**, 818–824.

BERLIN, N.I., NEUBERGER, A., SCOTT, J.J. (1956). The metabolism of δ-aminolaevulic acid. 1. Normal pathways, studied with the aid of $^{15}N$. *Biochem. J.*, **64**, 80–90.

CALDWELL, J. & MARSH, M.V. (1982). Metabolism of drugs by the gastrointestinal tract. In *Clinical Pharmacology and Therapeutics 1. Presystemic Drug Elimination*, George, C.F. & Shand, D.G. (eds), pp. 3–28. Butterworth Scientific: London.

CHAN, W.S., MACROBERT, A.J., PHILLIPS, D. & HART, I.R. (1989). Use of charged couple device for imaging of intracellular phthalocyanines. *Photochem. Photobiol.*, **50**, 617–624.

DAILEY, H.A. & SMITH, A. (1984). Differential interaction of porphyrins used in photoradiation therapy with ferrochelatase. *Biochem. J.*, **223**, 441–445.

DIVARIS, X.G., KENNEDY, J.C. & POTTIER, R.H. (1990). Phototoxic damage to sebaceous glands and hair follicles of mice after systemic administration of 5-aminolevulinic acid correlates with localised protoporphyrin IX fluorescence. *Am. J. Pathol.*, **136**, 891–897.

DOUGHERTY, T.J. (1990). Photodynamic therapy for the treatment of cancer: current status and advances. In *Photodynamic Therapy of Neoplastic Disease*, Kessel, D. (ed). Vol. 1, pp. 1–20. CRC Press: Boca Raton, Florida.

EDWARDS, S.R., SHANLEY, B.C. & REYNOLDSON, J.A. (1984). Neuropharmacology of delta-aminolaevulinic acid-I. Effect of acute administration in rodents. *Neuropharmacology*, **23**, 477–481.

EL-SHARABASY, M.M.H., EL-WASEEF, A.M., HAFEZ, M.M. & SALIM, S.A. (1992). Porphyrin metabolism in some malignant diseases. *Br. J. Cancer*, **65**, 409–412.

GENECIN, P. & GROSZMANN, R.J. (1991). Hepatic blood flow, measurement, and physiological regulation. In *Oxford Textbook of Hepatology*, IcIntyre, N., Benhamou, J.P., Bircher, J., Rizzetto, m. & Rodés, J. (eds), pp. 31–37. Oxford University Press: Oxford.

GEORGE, C.F. & SHAND, D.G. (1982). Presystemic drug metabolism in the liver. In *Clinical Pharmacology and Therapeutics 1. Presystemic Drug Elimination*, George, C.F. & Shand, D.G. (eds), pp. 3–28. Butterworth Scientific: London.

GOMER, C.J. & DOUGHERTY, T.J. (1979). Determination of [$^3H$]- and [$^{14}C$]-hematoporphyrin derivative in malignant and normal tissue. *Cancer Res.*, **39**, 146–151.

KENNEDY, J.C., POTTIER, R.H. & PROSS, D.C. (1990). Photodynamic therapy with endogenous protoporphyrin IX: basic principles and present clinical experience. *J. Photochem. Photobiol. B: Biol.*, **6**, 143–148.

LOH, C.S., BEDWELL, J., MACROBERT, A.J., KRASNER, N., PHILLIPS, D. & BOWN, S.G. (1992). Photodynamic therapy of the normal rat stomach: a comparative study between di-sulphonated aluminium phthalocyanine and 5-aminolaevulinic acid. *Br. J. Cancer*, **66**, 452–462.

LOH, C.S., VERNON, D.I., MACROBERT, A.J., BEDWELL, J., BOWN, S.G. & BROWN, S.B. (in press). Endogenous porphyrin distribution induced by 5-aminolaevulinic acid in the tissue layers of the gastrointestinal tract. *J. Photochem. Photobiol. B: Biol.*

MUSTAJOKI, P., TIMONEN, K., GORCHEIN, A., SEPPÄLÄINEN, A.M., MATIKAINEN, E. & TENHUNEN, R. (1992). Sustained high plasma 5-aminolaevulinic acid concentration in a volunteer: no porphyric symptoms. *Euro. J. Clin. Invest.*, **22**, 407–411.

POPE, A.J., MACROBERT, A.J., PHILLIPS, D. & BOWN, S.G. (1991). The detection of phthalocyanine fluorescence in normal rat bladder using sensitive digital imaging microscopy. *Br. J. Cancer*, **64**, 875–879.

RAZUM, N., BALCHUM, O.J., PROFIO, E. & CARSTENS, F. (1987). Skin photosensitivity: duration and intensity following intravenous hematoporphyrin derivatives, HpD and DHE. *Photochem. Photobiol.*, **46**, 925–928.

REDDI, E. & JORI, G. (1988). Steady-state and time-resolved spectroscopic studies of photodynamic sensitisers: porphyrins and phthalocyanines. *Chem. Intermediates*, **10**, 241–268.

RENWICK, A.W. (1982). First-pass metabolism within the lumen of the gastrointestinal tract. In *Clinical Pharmacology and Therapeutics 1. Presystemic Drug Elimination*, George, C.F. & Shand, D.G. (eds), pp. 3–28. Butterworth Scientific: London.

SARDESAI, V.M., WALDMAN, J. & ORTEN, J.M. (1964). A comparative study of porphyrin biosynthesis in different tissues. *Blood*, **24**, 178–186.

SCHOENFELD, N., EPSTEIN, O., LAHAV, M., MAMET, R., SHAKLAI, M. & ATSMON, A. (1988). The heme biosynthetic pathway in lymphocytes of patients with malignant lymphoproliferative disorders. *Cancers Lett.*, **43**, 43–48.

SZEIMIES, R.M., SASSY, T. & LANDTHALER, M. (1992). Studies on penetration depth in photodynamic therapy of basal cell carcinoma (BCC) with topical delta-aminolevulinic acid (ALA) (Abstract). *Lasers Med. Sci.*, **7**, 290.

TRALAU, C.J., BARR, H., SANDEMAN, D.R., BARTON, T., LEWIN, M.R. & BOWN, S.G. (1987). Aluminium sulfonated phthalocyanine distribution in rodent tumours of the colon, brain and pancreas. *Photochem. Photobiol.*, **46**, 777–781.

VAN DYKE, R.W. (1989). Mechanism of digestion and absorption of food. In *Gastrointestinal Disease, Pathophysiology, Diagnosis, Management*, 4th Edition, Sleisenger, M.H. & Fordtran, J.S. (eds), Vol. 2, pp. 1062–1088. W.B. Saunders: Philadelphia.

VAN HILLEGERSBERG, R., VAN DEN BERG, J.W., KORT, W.J., TERPSTRA, O.T. & WILSON, J.H.P. (1992). Selective accumulation of endogenously produced porphyrins in a liver metastasis model in rats. *Gastroenterology*, **103**, 647–651.

WEISHAUPT, K.R., GOMER, C.J. & DOUGHERTY, T.J. (1976). Identification of singlet oxygen as the cytotoxic agent in the photoactivation of a murine tumour. *Cancer Res.*, **36**, 2326–2329.